# Implementation of the Medication Literacy Assessment Instrument MELIA in the Home Care Setting: A Multi‐Method Study

**DOI:** 10.1002/nop2.70783

**Published:** 2026-08-26

**Authors:** Samuel Stenz, Franziska Zúñiga, Kornelia Kotkowski, Carla Meyer‐Massetti

**Affiliations:** ^1^ Department Public Health Institute of Nursing Science, University of Basel Basel Switzerland; ^2^ Practice Development Unit Spitex Basel Basel Switzerland; ^3^ Nursing Homes of the City of Zurich Zurich Switzerland; ^4^ Institute for Primary Healthcare (BIHAM), University of Bern Bern Switzerland; ^5^ Clinical Pharmacology and Toxicology, Clinic for General Internal Medicine Inselspital–University Hospital of Bern Bern Switzerland

**Keywords:** assessment instrument, home care organization, implementation science, medication literacy, nurses, nursing, older patients

## Abstract

**Aims:**

This study aimed to identify determinants for successfully implementing the MEdication LIiteracy Assessment tool (MELIA) by nurses, from developing an implementation plan to evaluating its feasibility in two Swiss home care organizations.

**Design:**

A multi‐center pilot study using a multi‐method design.

**Methods:**

Phase 1 (pre‐implementation) involved forming nurse stakeholder groups at each site to identify determinants of MELIA implementation, including potential barriers and facilitators. Subsequently, nurse stakeholders developed implementation plans with strategies to address identified determinants and to test MELIA within their organizations. In Phase 2 (pilot implementation), one care team per organization tested MELIA. Registered nurses (RNs) completed an anonymous questionnaire collecting focus on perceived acceptability and feasibility, with optional free‐text responses. Phase 3 consisted of focus group discussions with RNs from each organization to explore their experiences using MELIA, particularly regarding feasibility, acceptability, and adoption.

**Results:**

Two home care organizations participated. The stakeholder groups consisted of 1 team leader, 1 clinical nurse specialist, and 2 RNs at one site and 3 advanced practice nurses at the other site. Pilot testing was performed by seven and six nurses, respectively. Several barriers to the use of MELIA were identified by participating RNs: limited nurse–patient communication skills, insufficient knowledge about assessment tools, and unclear understanding of RNs' roles in structured assessments within interprofessional teams. Not all RNs were willing to conduct structured assessments, mostly influenced by awareness of patient benefits. Interprofessional collaboration was hindered by unclear communication and differing perceptions of role responsibilities. Organization and planning were time‐consuming, and some RNs were reluctant to read the lengthy instruction manual required for correct use.

**Conclusion:**

The study identified contextual factors influencing implementation of clinical interventions in home care. Key facilitators included RNs' awareness of MELIA's patient benefits, clear and accessible instructions, and defined professional roles in structured assessments and communication. Policymakers should strengthen treatment networks to enhance collaboration in medication management. Nursing education should emphasize communication skills and structured assessment instruments, while home care managers should provide clear structures and role guidance. Future research should examine transferability, scalability, and broader adoption of MELIA in home care organizations.

**Implications for the Profession and/or Patient Care:**

Combining clinical supervision with more education about using structured assessment instruments in general should increase RNs' knowledge and skills. This should support the desired behavioural change of having RNs use structured instruments more frequently to screen and assess the medication literacy of older adult home care patients.

**Impact:**

This study investigated the determinants of successfully implementing and using the MELIA tool. The identified barriers to and facilitators of this—primarily RNs' awareness of its benefits to patients, an easy‐to‐understand instruction manual, and RNs' communication skills regarding medication—can be used by policymakers, in nursing curricula, and in clinical practice. The study's insights will aid the implementation and use of the MELIA intervention in other home care settings, enabling the structured screening and assessment of older adult patients' medication literacy. This should subsequently lead to improved medication safety for older adult home care patients.

**Reporting Method:**

This multi‐method study applied the Standards for Reporting Qualitative Research guidelines.

**Patient or Public Contributions:**

Stakeholder participation was an integral part of this pilot implementation study. A stakeholder group was formed at each organization's headquarters, comprising professionals in leadership positions and nurses providing everyday care. These stakeholders provided contextual information and planned pilot testing by their home care organization. They also recruited the focus group interviewees at each headquarters.

**Trial and Protocol Registration:**

The protocol for this manuscript was not registered.

## 1 Introduction

Inappropriate medication management can lead to medication‐related problems (MRPs) (Parekh et al. 2018; Meyer‐Massetti, Hofstetter, et al. 2018), a concept combining adverse drug reactions and medication errors (Nebeker et al. 2004). Adverse drug reactions are defined as harm caused by appropriately used drugs; medication errors are defined as inappropriate drug use that may cause harm. Near misses are potential medication errors that were detected before they caused harm (Nebeker et al. 2004). MRPs are frequent in home care settings, affecting between 19.8% and 48.4% of prescribed drugs, and up to 26% of these were considered harmful medication errors (Meyer‐Massetti, Meier, and Guglielmo 2018). The most frequently reported risk factors for MRPs were polypharmacy (taking > 9 drugs) and increasing age (Meyer‐Massetti, Meier, and Guglielmo 2018; Godfrey et al. 2013). MRPs involving older adults can be linked to medication management problems. In home care settings, patients and informal caregivers play important roles in medication management processes and maintaining medication safety (Meyer‐Massetti, Meier, and Guglielmo 2018; Nicosia et al. 2020).

Older adults describe a wide array of medication management problems, which can be divided into three categories (Nicosia et al. 2020). Firstly, *problems taking medication* encompass problems with the adequate application of medication, i.e., the practical aspects of drug preparation, using the correct dose and using it according to the prescribed frequency and schedule (Nicosia et al. 2020). Secondly, *problems with medication's effects* refer to adverse drug reactions (Nebeker et al. 2004). Older adults are prone to this kind of problem because advanced age can lead to pharmacodynamic and pharmacokinetic changes, potentially making them more susceptible to adverse drug reactions (Parekh et al. 2018). Thirdly, *problems with communication, care coordination, and medication information*. Communication is a key factor in effective medication management at both the patient and healthcare system levels. Older adults describe the importance of communication in being able to trust their healthcare provider (Nicosia et al. 2020). Besides older adults themselves, medication management processes often involve multiple professionals working in different settings, such as general practitioners (GPs), registered nurses (RNs), and pharmacists (Godfrey et al. 2013). Multiple interfaces require more coordination, which can also increase the risk of errors (Godfrey et al. 2013).

**FIGURE 1:**
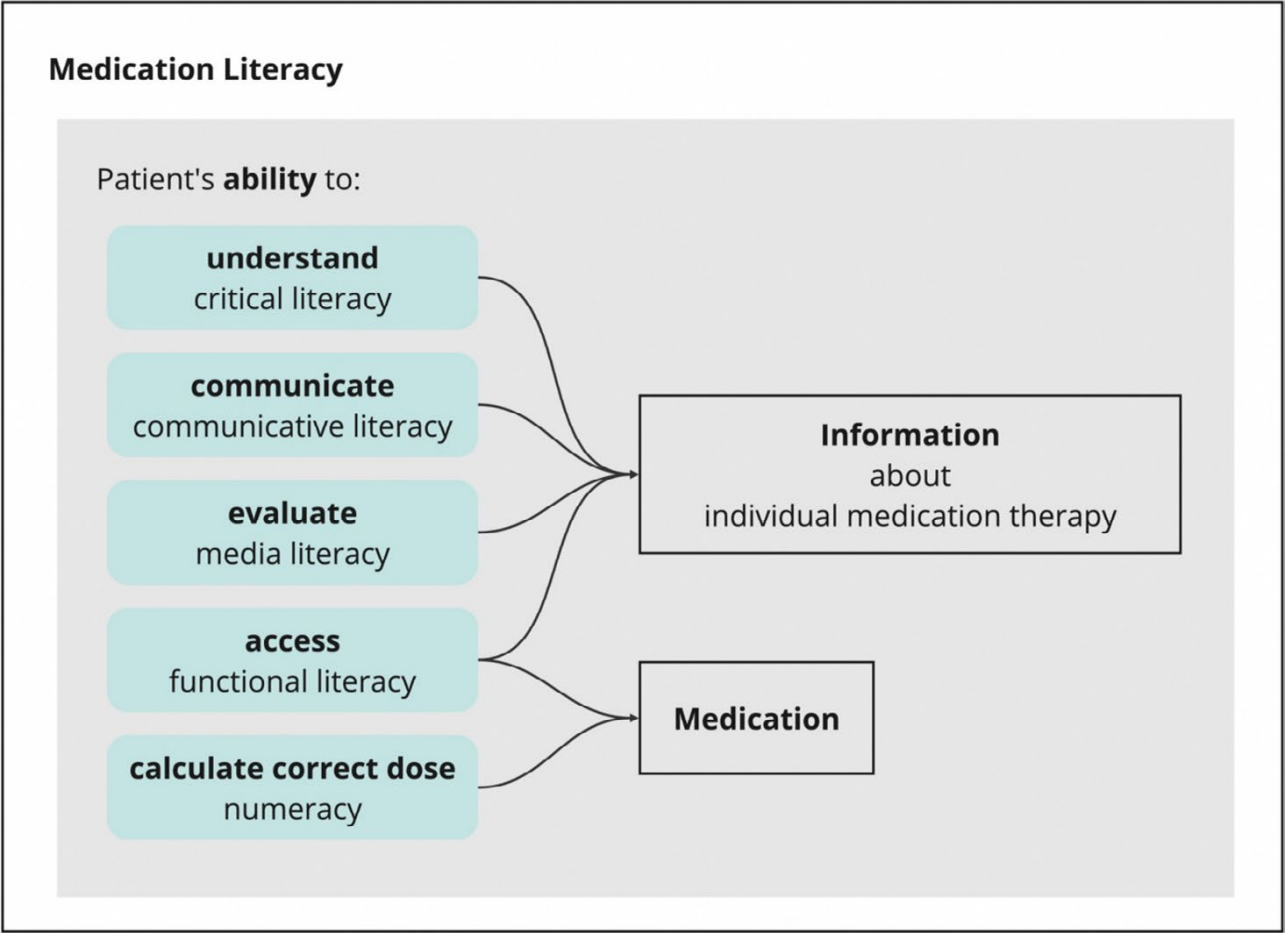
The five dimensions of medication literacy.

Adequate medication management requires medication literacy, a multidimensional concept consisting of a patient's ability to communicate, obtain, process, comprehend, and calculate information about each individual medication (Pouliot et al. 2018; Neiva Pantuzza et al. 2022; Pantuzza et al. 2020). Figure 1 illustrates its five dimensions.

Professional care for a polymedicated older adult should include an assessment of their capacity to self‐manage their medication (Liau et al. 2021). Reliable, evidence‐based instruments are needed to systematically screen and assess medication literacy among older adults with multimorbidity and polypharmacy, as they have an increased risk of inadequate medication literacy (Plaza‐Zamora et al. 2020). A systematic review by Gentizon et al. (2021) identified 13 instruments, but only the 14‐item MedLitRxSE assessment tool was recommended for clinical use in the care of adult patients (Gentizon et al. 2021). However, MedLitRxSE does not measure the ability to communicate information or act upon it, which is the essence of medication management (Gentizon et al. 2021).

The MEdication LIteracy Assessment (MELIA) instrument was developed specifically to help RNs assess home‐dwelling older adult (≥ 65) patients' medication literacy in home care settings in Switzerland and, ultimately, to optimize medication safety (Gnagi et al. 2022). Its short version contains seven items for use by RNs working for professional home care organizations to identify individuals at a high risk of medication management problems (Gnagi et al. 2022). The extended, 20‐item version of the MELIA should be used for in‐depth analyses of older adult home care patients' medication literacy after the short version has indicated a potential problem (Gnagi et al. 2022). Little is currently known about how interventions to introduce medication literacy instruments, like the MELIA, can be successfully implemented in home care settings. To the best of our knowledge, no studies focusing on this specific question have been published.

### 1.1 Study Goals

This multi‐method study's primary goal was to describe the determinants affecting the implementation of the MELIA, with a dual focus on the development of an implementation plan and the evaluation of the instrument's use by two Swiss home care organizations.

Specific study aims were:

1. To develop implementation plans for the MELIA tool for two home care organizations in Switzerland, in collaboration with key stakeholders:
  - to explore the context surrounding the use of the MELIA in home care settings, to describe the determinants (i.e., barriers and facilitators) of the planned implementation on the target behaviour of RNs screening and assessing medication literacy;
  - to develop implementation strategies to address the identified barriers to and facilitators of using the MELIA from the perspective of RNs.
2. For two home care organizations to implement the MELIA during a feasibility pilot project and test the implementation plan developed.
3. To evaluate the MELIA's implementation plan and use based on the outcomes of its acceptability, feasibility, and adoption from the perspective of RNs.

## 2 Methods

### 2.1 Design

**FIGURE 2:**
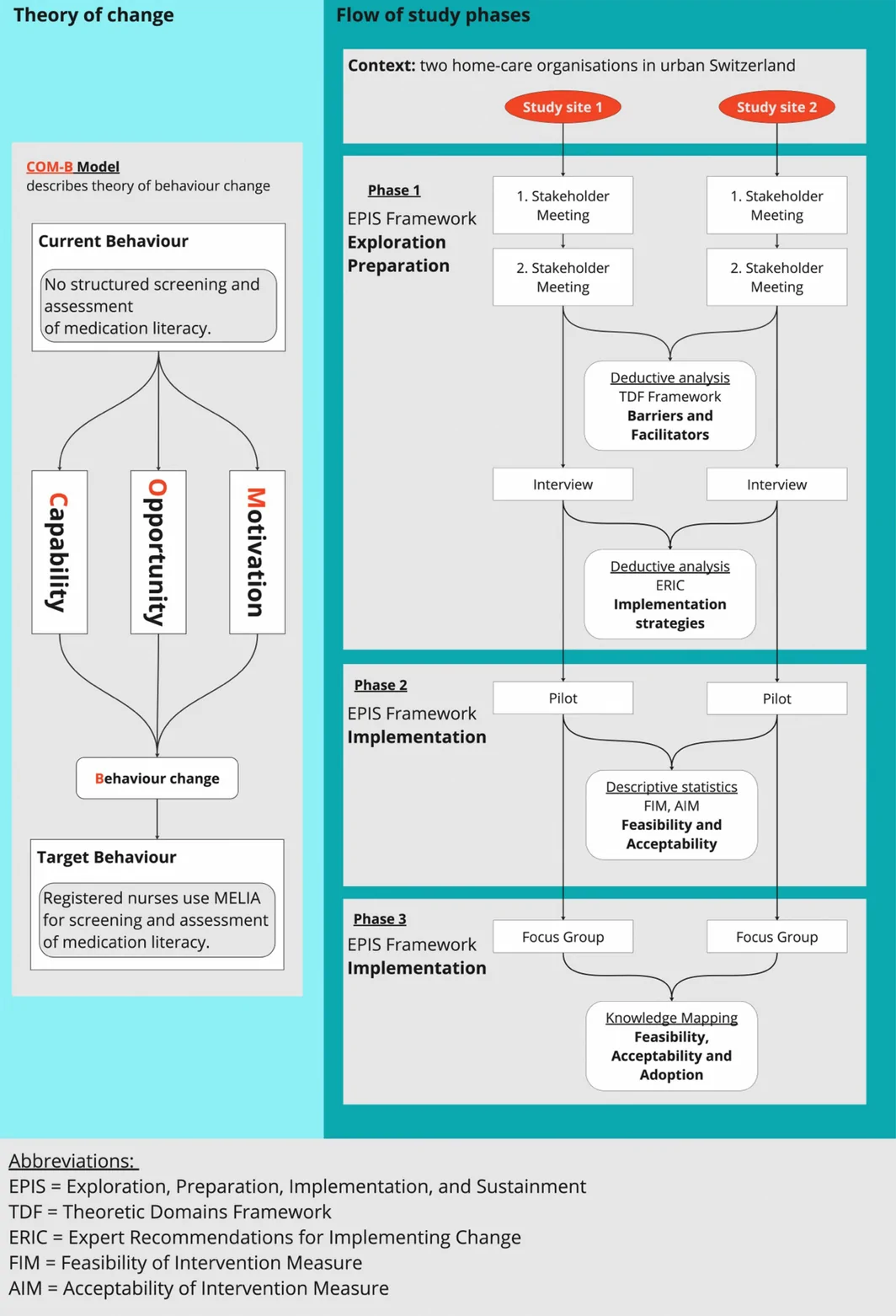
Multi‐method design: Methods for data collection and data analysis approaches.

The study followed a multi‐method design, with a multi‐center project setup. The design was based on the first three phases described in the Exploration, Preparation, Implementation, Sustainment (EPIS) framework (Aarons et al. 2011). Figure 2 illustrates the study design. A detailed description of each phase of the study is given below.

#### 2.1.1 Phase 1—Exploration and Preparation (Pre‐Implementation)

Phase 1 involved exploring potential barriers to and facilitators of implementation, as well as developing an implementation plan with key stakeholders. Stakeholder groups were formed at two sites of each organization. An initial meeting identified potential barriers to and facilitators of implementing the MELIA and ideas for possible implementation strategies.

A second meeting developed those implementation strategies to address the barriers and facilitators. The research team helped the stakeholders to tailor their implementation strategies to overcome the barriers identified, and they acted as implementation advisors in accordance with the Expert Recommendations for Implementing Change (ERIC) project (Powell et al. 2015). Stakeholders at each organization's study site then further developed and implemented specific strategies for using the MELIA. Interviews were conducted with one member of each stakeholder group to identify the implementation strategies actually used at each study site. Table 1 gives an overview of the study participants.

**TABLE 1:** An overview of the study participants.

Type	Study site 1	Study site 2
Stakeholder groups	Team leader (*n* = 1) Clinical nurse specialist (*n* = 1) RN/Case manager (*n* = 1) RN (*n* = 1)	Advanced practice nurses (*n* = 3)
Interviewees	RN/case manager (*n* = 1)	Advanced practice nurse (*n* = 1)
Pilot testing	RNs (*n* = 6)	RNs (*n* = 7)
Focus groups	RNs (*n* = 2) RN/practice instructor (*n* = 1)	RNs/case managers (*n* = 3)

#### 2.1.2 Phase 2—Pilot Test Implementation

Phase 2 tested the implementation plan developed within the scope of a feasibility pilot test. RNs working at the two study sites were asked to use the MELIA at least twice. This phase's goals were to test the MELIA's use in real‐life conditions and measure the implementation outcomes of acceptability and feasibility (Proctor et al. 2011).

#### 2.1.3 Phase 3—Pilot Test Evaluation

After Phase 2's pilot testing, focus group (FG) interviews were conducted with the RNs who tested the MELIA. These Phase 3 interviews aimed to explore participants' experiences of using the MELIA during pilot testing and to gather more contextual information on what might lead to the instrument's acceptability, feasibility, and adoption.

#### 2.1.4 Rationale

The study's overall rationale followed a critical realist approach, which treats reality as a social construct and suggests it is impossible for human beings to fully replicate the real world. Claims about participants' existing knowledge must, therefore, always be bounded by context, and the circumstances under which the explanations are likely to hold must be clarified (McEvoy and Richards 2016; Schiller 2016). Within this philosophical framework, it is assumed that human actors respond differently to interventions under different circumstances.

### 2.2 Theoretical Framework

This study was based on the programme logic that behavioural change is inherent when evidence is translated into practice (Handley et al. 2016). This theory of change is explained within the COM‐B model, which is itself based on the observation that a new behaviour will only occur if the capability, opportunity, and motivation to engage in a certain behaviour are present (Kim et al. 2020).

To help classify the underlying determinants of project implementation, the present study used the Theoretical Domains Framework (TDF), which consists of 12 theoretical domains, to assist the theoretical assessment of implementation issues (Cane et al. 2012). The 12 theoretical domains can be linked to capability, opportunity, and motivation, as described in the COM‐B model (Atkins et al. 2017). To ensure an adequate description of the barriers to and facilitators of implementation, using consistent language, we used the published compilations of terms and definitions from the ERIC project (Powell et al. 2015).

### 2.3 Study Setting and Recruitment

#### 2.3.1 Contextual Information and Study Setting

There are 2200 home care organizations in Switzerland, known as ‘Spitex’, a German acronym for “hospital‐external care”. These employ approximately 52,000 home care professionals overall (Schur et al. 2020). Two non‐profit home care organizations in different cities in the German‐speaking regions of Switzerland voluntarily pilot‐tested the MELIA's feasibility.

Study Site 1, organized into six districts, was covered by a non‐profit trust with a cantonal service mandate. In 2023, it supported 4606 clients, 51% of whom were aged 80 or older, and it employed 586 staff in the equivalent of 412 full‐time positions. Study Site 2, organized into ten districts, was covered by a self‐organized non‐profit organization with a service mandate from a city. In 2023, it supported 2135 clients, 50.7% of whom were aged 80 or older, and it employed 322 staff in the equivalent of 225 full‐time positions.

In home care settings in Switzerland, medication is mostly prescribed by GPs who practice independently from home care organizations. In some cases, specialized physicians or hospitals are also involved in medication prescription processes. Differing cantonal laws regulate whether medications must be dispensed by a pharmacy or whether GPs in so‐called “self‐dispensing practices” can dispense them directly to their patients. Study Site 1 was located in a pharmacy‐dispensing canton, and study Site 2 was located in a self‐dispensing and pharmacy‐dispensing canton. These factors have led to a complex national environment for managing patients' medications, and home care organizations are mandated with the task of coordinating patient medication‐use processes.

#### 2.3.2 Study Site Recruitment Process and Sampling Strategy

Two home care organizations suitable for the study were identified using purposeful sampling and the research team's knowledge of them. Non‐committal email invitations were sent to individuals with leadership positions in the organizations chosen. Both home care organizations stated their provisional interest in taking part in the study and were sent an official email request to participate, accompanied by a short information sheet explaining the project.

#### 2.3.3 Phase 1 Recruitment Process (Stakeholder Meetings and Interviews)

One stakeholder group was formed for each home care organization, including individuals in leadership positions and RNs providing direct patient care.

To form study Site 1's stakeholder group, the first author initially presented the study to the organization's management board, which then assigned one of its six districts to participate. The district team leader sought interested staff from within her team to join the group. The stakeholder group finally comprised four members with different roles within the team.

After the executive board of the second home care organization had agreed to participate in the study, Site 2's stakeholder group was formed directly from a team of advanced practice nurses fulfilling the combined roles of practice development experts and providers of direct clinical care.

One member from each stakeholder group was recruited for an interview—an individual who had an overview of the entire pilot implementation strategy applied by their home care organization. Stakeholder group and interview inclusion criteria are shown in Table 2.

**TABLE 2:** Study phase and study site inclusion criteria.

	Inclusion criteria
Study sites	Home care organization employing RNs Home care organization assisting polymedicated older adults (≥ 65) with managing their medication Located in the German‐speaking part of Switzerland
Phase 1	Stakeholder group Stakeholders employed by home care organizations participating in the study Heterogeneity through the inclusion of participants in leadership positions and RNs providing direct clinical care Interview Stakeholder group members who were part of the entire implementation process
Phase 2	Participants to complete the questionnaire RNs who used the MELIA in pilot testing Able to speak and read German
Phase 3	Focus group interviewees RNs who used the MELIA in pilot testing Able to speak and read German

#### 2.3.4 Phase 2 Recruitment Process (Pilot)

The MELIA was tested in one care district of each participating home care organization's area of operations. All the RNs working in this district were asked to participate in the study. At study Site 1, RNs were asked by their team leader; at study Site 2, they were asked by an advanced practice nurse.

#### 2.3.5 Phase 3 Recruitment Process (Focus Group Interviews)

FG interviewees were recruited by members of their respective stakeholder groups. They then received a written invitation with study information and an informed consent form from the research team.

### 2.4 Inclusion Criteria

See Table 2.

### 2.5 Data Collection

**FIGURE 3:**
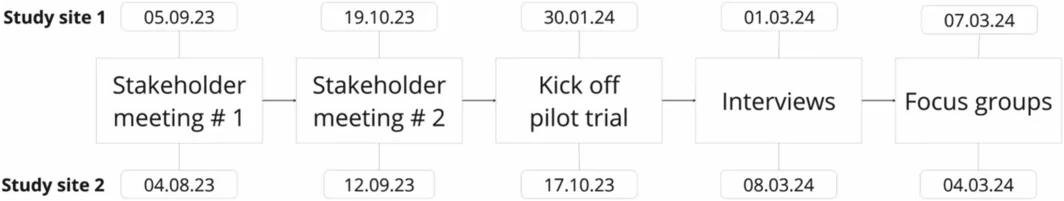
Qualitative data collection timeline.

The following paragraphs describe each study phase's data collection procedures. Table 3 gives an overview of the qualitative data collection process, and Figure 3 shows the timeline for qualitative data collection.

**TABLE 3:** Qualitative data collection process.

Event	Process	
Stakeholder meeting #1	Participants got to know each other Study introduced and meeting's goals explained Potential barriers to and facilitators of using the MELIA to screen and assess patients' medication literacy (target behaviour for RNs) were identified First ideas for implementation strategies collected	
Stakeholder meeting #2	Review of findings from stakeholder meeting #1 conducted with the study participants Implementation plan developed based on potential determinants (barriers and facilitators) identified in stakeholder meeting #1	
Interview	Identification of the implementation strategies applied by each home care organization	
Pilot testing	Interviewees completed questionnaires about using the MELIA, with an option to leave their comments	
Focus group	Interviewees' perspectives on the MELIA's acceptability, feasibility, and adoption were explored	

Abbreviations: MELIA, medication literacy assessment; RN, registered nurse.

#### 2.5.1 Phase1

The first stakeholder meeting for study Site 1 was organized by its team leader, and a second meeting was scheduled during that. The three stakeholders at study Site 2 organized their first meeting together, and a second meeting was also scheduled during that time. Stakeholder meetings took place at each organization's site and were led by the first author; the last author took meeting minutes. Participants were asked to write answers on sticky notes, which were then clustered on flip charts, and at the end of each 1.5‐h meeting, photographs were taken of the clusters.

One‐on‐one interviews were also held at the end of Phase 2 at each study site. The two participants received an email invitation in advance, which included relevant study information. The first author performed the interviews and audio‐recorded them. At study Site 1, the interview took 28 min; at study Site 2, it lasted 34 min.

#### 2.5.2 Phase2

RNs were asked to complete a three‐section questionnaire after each use of the MELIA, including: (a) demographic data of the healthcare professional themselves (age, gender, years worked in the home care organization, and whether they had used the MELIA before); (b) the German version of the acceptability of the intervention measure (AIM) (Weiner et al. 2017; Kien et al. 2021) and the German version of the feasibility of the intervention measure (FIM) (Weiner et al. 2017; Kien et al. 2021); and (c) a space for comments and free‐text answers. The questionnaire is available in Appendix 1. Data were entered manually into an MS Excel software spreadsheet (Version 16.85).

#### 2.5.3 Phase 3

FGs were organized by each home care organization's stakeholder group. FG participants received an email invitation in advance, including relevant study information from the research team. The FGs were held at each organization's site and were audio‐recorded. They took 85 min at study Site 1 and 45 min at study Site 2. During each focus group, the first author drew knowledge maps and reflected upon them with the participants, while the last author took notes.

### 2.6 Data Analysis

#### 2.6.1 Phase 1

Theory can guide behaviour change interventions, and the classification of the determinants of that intervention's implementation can help us better understand the implementation's context (Cane et al. 2012; Atkins et al. 2017). Thus, guided by the TDF framework, we applied a deductive approach to analysing our data to identify the key determinants influencing the MELIA's implementation and ERIC to pinpoint potential implementation strategies (Powell et al. 2015; Atkins et al. 2017; Gale et al. 2013).

#### 2.6.2 Phase 2

Quantitative data from the questionnaires were analysed by computing descriptive statistics using R Studio software (Version 2023.12.1). These included sample size, central tendencies (mean, median and mode), standard deviations, and minimum and maximum values.

#### 2.6.3 Phase 3

In Phase 3, we evaluated the MELIA's implementation. Data analysis started during our FGs using knowledge mapping of the participants' reflections (Burgess‐Allen and Owen‐Smith 2010). This rapid qualitative data analysis method can be used to model participants' ideas, beliefs, values, and attitudes. It can illustrate many kinds of relationships (e.g., interpersonal or interorganizational) and has the advantage of following natural thinking patterns, making it ideal for use in studies involving stakeholder participation (Burgess‐Allen and Owen‐Smith 2010). Each FG's knowledge map was refined after listening to their respective audio recordings, and a meta map was drawn, combining the insights from both the home care organizations' study sites into one comprehensive map (Burgess‐Allen and Owen‐Smith 2010). Phase 3's final analytical step was describing the knowledge depicted in the meta map (Tessier 2012).

### 2.7 Ethical Considerations

#### 2.7.1 Ethical Issues Pertaining to Human Subjects

The Ethics Commission of Northwestern and Central Switzerland (EKNZ) verified the research proposal and concluded that the study was a quality improvement project and therefore did not require ethics approval as it did not fall under the scope of the Federal Act on Research involving Human Beings (Req‐2023‐00741).

The research participants were informed about the study in advance, and they provided their written consent before their first one‐to‐one interview and at the start of each FG meeting. Project data were handled with the utmost discretion and were only accessible to authorized research team members who required the data to fulfil their duties within the scope of the research project. On any project‐specific documents, participants were only identifiable by their unique participant number. Quantitative data were collected anonymously; individual data sheets could not be traced back to individual participants. Participants were appropriately informed and agreed that data could be published in an anonymized format.

### 2.8 Rigour and Reflexivity

#### 2.8.1 Researcher Characteristics and Reflexivity

The first author was also employed by one of the participating home care organizations as an RN. During the study, his position changed to that of a nursing expert. He had never worked in the district where pilot testing was conducted. Before the study, the senior author had once worked for the other home care organization as a consulting pharmacist.

They used a participatory research approach, with the main author leading the stakeholder meetings and the last author writing the minutes. Decisions concerning implementation processes were always made by stakeholders from the respective home care organization.

#### 2.8.2 Techniques to Enhance Trustworthiness

The knowledge maps drawn during the FGs were reviewed with participants, allowing them to provide feedback if a researcher had misunderstood what they were trying to explain. The co‐authors checked the initial codes representing key themes emerging from the knowledge maps and discussed them with peers who were not employed by the home care organizations.

## 3 Findings

### 3.1 Study Participants

See Table 1.

### 3.2 Study Results

#### 3.2.1 Phase 1

Based on the stakeholder meetings, a list of the potential determinants of the successful intervention implementation and use of the MELIA, in the form of barriers and facilitators, was created using the COM‐B model, in combination with the TDF. Themes were described using domain summaries (Kim et al. 2020; Cane et al. 2012; Atkins et al. 2017). The complete list is available in Appendix 2.

Regarding capability, stakeholders emphasized knowledge management and training and guidance. Stakeholders proposed that RNs be given an opportunity to share new knowledge and receive training before they conduct the MELIA for the first time. They also proposed guided processes as important for the successful implementation of the MELIA intervention.

Regarding opportunity, stakeholders emphasized internal and external processes and noted that RNs needed to know what must be done by whom and at what time to successfully conduct the structured screening and assessment of medication literacy. They also proposed that asking the MELIA's questions should be as easy as possible.

Regarding motivation, stakeholders emphasized beliefs about consequences: RNs should know about the potential benefits a structured assessment using the MELIA brings to patients. They also deemed it important that RNs have a clear understanding of their roles and responsibilities in the successful use of the MELIA.

**FIGURE 4:**
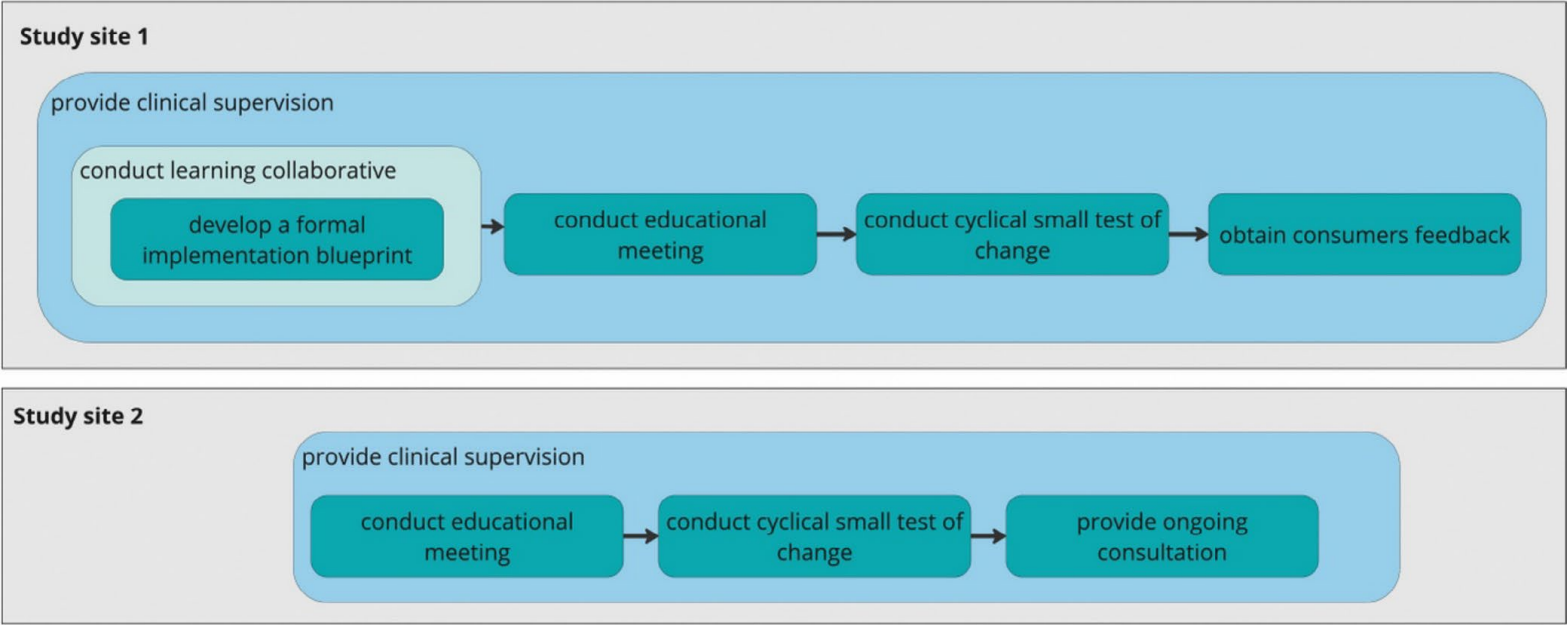
Overview of the implementation strategies as categorized and structured in the Expert Recommendations for Implementing Change (ERIC) project.

The stakeholder groups at each study site developed slightly different implementation strategies for facilitating the behaviour change needed to introduce the regular, structured screening and assessment of medication literacy. The strategies to be applied at each study site were identified via one‐to‐one interviews with one stakeholder per group and are displayed in Figure 4.

The stakeholder group at study Site 1 planned to start their implementation process by forming a learning collective consisting of additional case managers and the original stakeholder group members. The learning collective's members met twice before pilot testing began. The first meeting discussed the right approach to implementation, collected initial ideas for a formal implementation blueprint, and the collective's members decided to familiarize themselves with the MELIA. The first meeting was informal, and no minutes were taken.

The second meeting, organized by a case manager rather than the team leader, was more formal, and decisions were documented with meeting minutes. The meeting formalized the implementation blueprint. The learning collective reflected on its conclusions and questions that had appeared during its familiarization with the MELIA. The collective also focused on how RNs should ask their patients the MELIA's questions.

At both study sites, pilot testing started with an initial training session for the RNs. At study Site 2, this training also included a discussion about patients for whom the MELIA might be applied. Stakeholders from both study sites described RNs' apparent interest in the instrument during their training, and each RN was given the responsibility of assessing the medication literacy of at least two different patients using the MELIA.

The practical test of the intervention was conducted in limited sequential tests at both study sites. Study Site 1's stakeholder group assigned these tests to RNs without any experience in case management. At study Site 2, however, RNs' everyday responsibilities also included some case management tasks.

Study Site 1 planned a meeting to evaluate the pilot testing and to collect staff feedback, but this was cancelled. One interview participant attributed this to a shortage of staff (e.g., due to sickness), vacations, local public holidays and the high workload of employees in leadership positions.

More than 1 month after the start of pilot testing, however, no MELIA had been performed at either study site. RNs in both only started to use the MELIA after one person from each stakeholder group took the lead and assigned assessments of particular patients to particular RNs. This might also have been facilitated by members of the research team, who had requested an update on the current state of the pilot testing via email.

The study sites were organized differently. Study Site 1 had a hierarchical structure with RNs under team leaders; at study Site 2, RNs were self‐organized. This seemed not to influence the implementation process, however. At both study sites, the MELIA was only used after one person took the lead. In the organization with a hierarchical structure, it was not a team leader who assigned this dedicated person, but a case manager who took the lead on her own.

#### 3.2.2 Phase 2

Using the MELIA was tested by one team from each home care organization. After each MELIA, the RN applying it completed a questionnaire on its acceptability and feasibility (Proctor et al. 2011). Of the 13 RNs who tested the MELIA, ten (77%) were female, two (15%) were male, and one (7%) preferred not to answer the question concerning their gender. The mean participant was 36.5 years old (min = 24, max = 64, SD = 11.1) and had worked in home care settings for 5.3 years (min = 0.3, max = 12, SD = 3.5). The mean AIM score of 3.8/5 (IQR25 = 3, median = 4, IQR75 = 4.3) indicated that RNs found the intervention fairly acceptable to them. The mean FIM score of 4.2/5 (IQR25 = 4, median = 4, IQR75 = 4.8) indicated that RNs estimated that using the MELIA was highly feasible.

#### 3.2.3 Phase 3

**FIGURE 5:**
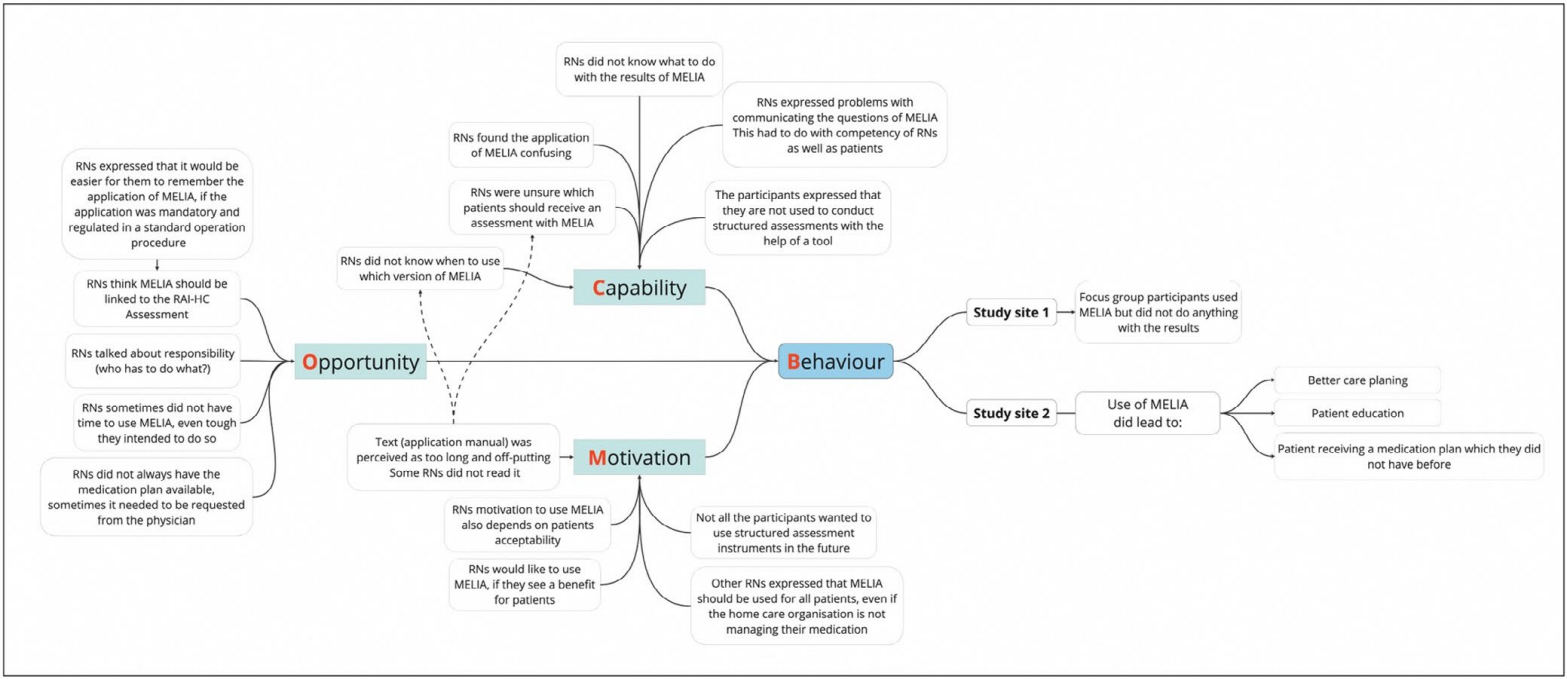
Knowledge map combining the results of the FG interviews at both study sites. RAI‐HC, Resident Assessment Instrument—Home Care Version; RN, registered nurse.

The two study site FGs used the knowledge mapping technique to add more insights into the RNs' views on the acceptability, feasibility, and adoption of the MELIA after pilot testing it (Burgess‐Allen and Owen‐Smith 2010). Their combined map is shown in Figure 5.

A list of the study's key findings is presented in Table 4. The following paragraphs describe the findings from our FGs, structured according to the COM‐B model and the domains of the TDF, linked to the determinants of the MELIA's successful implementation, e.g., the barriers and facilitators collected during stakeholder meetings.

**TABLE 4:** Key findings.

COM‐B	TDF	Key findings
Capability	Skills Knowledge	RNs' lack of skills in nurse–patient communication was identified as a barrier to using the MELIA. This lack of skills could also lead to the lower acceptability of structured assessments among patients Some RNs lacked knowledge about how to conduct assessments using questionnaires and lacked information about their roles' responsibilities and those of others
Opportunity	Social influence Environmental context and resources	Interprofessional cooperation challenged the participants due to unclear communication channels and different (self‐)perceptions about their roles' responsibilities At both study sites, implementation only started after someone proactively took the lead Organizing and planning assessments using the MELIA were suggested to be problematic. Time was needed to conduct and organize assessments, e.g., receiving medication plans from GPs
Motivation	Beliefs about consequences Intentions Social/professional role and identity	Awareness of the benefits to patients could motivate RNs to use the MELIA RNs were unwilling to read long texts to understand how to use an assessment instrument Not all the RNs were willing to conduct structured assessments of any kind Not all the RNs viewed themselves as members of a profession that conducted structured assessments

Abbreviations: GP, general practitioner; MELIA, medication literacy assessment; RN, registered nurse.

#### 3.2.4 Capability

##### 3.2.4.1 TDF Skills

Stakeholders reported lacking suitable abilities in empathetic communication, which became a potential barrier to the desired behaviour change, as they had insufficient skills to adequately ask questions. FG participants mentioned this, too, reporting difficulties in asking the MELIA's individual questions. They also mentioned their concerns that too much information about medication could lead to a decrease in patients' quality of life, e.g., if a patient prescribed anticoagulants was informed about the potential risks and subsequently stopped taking them. Others suggested that patients might perceive asking questions about medication as intrusive.

You have to think very carefully about how you ask the questions so that you don't intrude too much.

##### 3.2.4.2 TDF Knowledge

FG participants from one study site, who had never been involved in care planning, stated that they did not know when to do what and that the responsibilities for who was to conduct the MELIA should be clear. They explained that they lacked knowledge about what to do with the MELIA's results. The FG participants from the other study site, who were involved in care planning, did not mention these problems.

#### 3.2.5 Opportunity

##### 3.2.5.1 TDF Social Influence

Stakeholders stated that communication with other healthcare professionals could be a barrier to the desired behaviour change and the implementation of new interventions. Anecdotally, they described problems communicating with GPs, who only responded to home care nurses after repeated enquiries about patients' medication plans. Indeed, some stakeholders felt that some physicians did not take them seriously. One suggested that these negative experiences could be a barrier to the desired behaviour change because individual RNs might not pass on the results of the MELIA if they expected dismissive treatment from a physician or perceived communication with other healthcare professionals to be an administrative hassle. The FG participants, however, did not report experiencing problems with interprofessional communication during the pilot testing.

##### 3.2.5.2 TDF Environmental Context and Resources

In some cases, to use the MELIA correctly, FG participants first had to request a medication plan from the patient's GP. Before that, RNs also wanted to obtain their patients' consent. This and other administrative tasks linked to completing a MELIA were considered time‐consuming. The same was said about using the MELIA itself: one FG interviewee stated their surprise at how much time a correct assessment of medication literacy took, and they suggested that RNs new to using the MELIA should schedule enough time for it. The stakeholders also proposed time pressures as a potential barrier to using the MELIA; they found it important to ensure that RNs were aware that they could take the time needed because case managers usually factored in time reserves.

Another potential barrier to the desired behaviour change was poor usability. Stakeholders stated that the MELIA should be as easy to use as possible. FG participants at both study sites agreed that the accompanying manual was unusable in a long‐text format. In contrast, FG participants at the two study sites differed in their opinions on the MELIA's simplicity of use. One FG described it as clear and easy to use, but the other described it as unclear and difficult to use.

It was massive. Everything was so nicely color‐coded, but that also confused me a bit.

None of the FG participants reported knowing when to use the MELIA's different versions (short and extended), indicating that the manual was not working as intended.

Written instructions on using the MELIA correctly were identified as a barrier. FG participants stated that they perceived the explanatory text on using the MELIA to be too long and therefore off‐putting. They did not want to read long texts during their workday. Others just ignored the explanatory text completely. This led to knowledge gaps: none of the FG participants knew when to use each version of the MELIA.

#### 3.2.6 Motivation

##### 3.2.6.1 TDF Beliefs About Consequences

One FG participant stated that they would be willing to use the MELIA regularly if they saw clear benefits for patients. Stakeholders talked about this, too, mentioning that RNs' perceptions that interventions benefited patients were a potential facilitator of the target behaviour.

##### 3.2.6.2 TDF Intentions

Several stakeholders, from both study sites, intended to use the MELIA in the future; however, neither site had made clear plans on how or when to implement it. FG participants at the self‐managed study site were highly motivated to use the MELIA in the future and stated that it should be used with every older adult home care patient taking medication, even if the home care organization was not tasked with their medication management. Some FG participants from the other site seemed reluctant to use any kind of structured assessment instrument and thought that nurses should identify this kind of information by examining and talking freely with patients and each other without using a particular instrument.

Some RNs only used the MELIA after being told to do so by a case manager or an advanced practice nurse. Some FG participants stated that they were not motivated to use the MELIA and had only done so because of the study. Other healthcare professionals were highly motivated to use the MELIA. However, all participants had only started using the MELIA after a hierarchical superior had specifically tasked them to do so.

##### 3.2.6.3 TDF Social/Professional Role and Identity

FG participants in one study site discussed nurses' roles within medication processes and stated that they were not used to performing structured clinical assessments in the form of questionnaires. They mentioned that historically, nurses had not done such assessments. In the FG participants' opinions, these tasks were normally assigned to other professionals, such as psychologists or physicians. Not all the FG participants agreed that performing structured assessments should be a part of nursing care. Some thought they might use structured assessments in the future, but only if they did not take up too much of their time and used up < 30% of the workday. Stakeholders agreed on this and, consequently, suggested that using the MELIA could be facilitated if individual RNs knew it was part of their job and, therefore, also part of their role. It is worth mentioning that younger FG participants were less likely to question whether the use of structured assessments was a part of nursing care; they also seemed highly motivated to use the MELIA in the future.

So, doctors and psychologists work with these kinds of things a lot more, and nurses really just use the pain [scale], the Norton scale and, um, for pressure ulcers, yes, there aren't many. […] So, I think that we nurses are not really used to so many assessments of this type.

## 4 Discussion

The present study revealed new contextual information about how to conduct the MELIA in home care settings. We created a list of potential barriers to and facilitators of the behaviour change of having RNs use this structured assessment instrument with older adult home care patients. Quantitative results from pilot testing the MELIA showed good acceptability among RNs (mean AIM score = 3.8/5) and good feasibility (FIM mean score = 4.2/5) for implementing the intervention. Qualitative evaluation of the pilot testing revealed that RNs lacked the communication skills to explain the MELIA's items to the patients and lacked knowledge about how to use assessment instruments in general and what their responsibilities were once the information was gathered. Unclear communication channels and unclear perceptions about roles led to organizational problems in RNs' interprofessional cooperation with GPs. Less experienced RNs had problems with the MELIA's usability. RNs' motivation to conduct structured assessments depended on their awareness of the benefits to patients, easy‐to‐understand instruction manuals, and clarity about RNs' precise contribution to performing structured assessments within interprofessional teams and their role in medication management processes. The paragraphs below are structured according to the COM‐B model.

### 4.1 Capability

RNs' communication skills were identified as a barrier to using the MELIA appropriately. The way nurses communicated was suggested as a determinant of whether patients accepted undergoing an assessment. This was in line with an integrative review by Hoglander et al., which concluded that nurse–patient communication can affect clinical outcomes, e.g., medication adherence (Hoglander et al. 2023). Patients' communication styles are affected by the type of communication RNs use (Hoglander et al. 2023). RNs seem to use task‐oriented communication rather than asking questions to explore patients' doubts and using active listening techniques (Hoglander et al. 2023).

Capability was also linked to RNs' knowledge. Stakeholders at both study sites dedicated a portion of their implementation plan to training RNs how to correctly use the MELIA. Despite that, we identified interprofessional collaboration with unclear responsibilities as a barrier to implementing new interventions in home care settings. Stakeholders stated that they did not know who had to do what and when during assessing a home care patient's' medication literacy. This could be mitigated by clear organizational structures, with effective leadership enforcing the use of evidence‐based assessment instruments instead of just providing education and leaving RNs to their own devices. This was in line with a systematic review by Spoon et al., which concluded that education itself only had a moderate effect on guiding the implementation of evidence‐based practice (Spoon et al. 2020). According to the integrative review by Pursio et al. (2021), it falls to nurses in leadership positions to ensure that RNs know what the expectations and responsibilities of their role are. Indeed, they should be supported through an environment where these expectations and responsibilities are the same in every situation.

Overall, the literature supports our findings that educational means need to be combined with facilitation and organizational changes (Powell et al. 2015; Spoon et al. 2020).

Another important aspect, linking capability and opportunity, is communication, ideally facilitating acceptance of assessment activities by patients. Höglander et al. stressed that communication is a multifaceted process encompassing diverse strategies that are fundamental to establishing trust and facilitating patient‐centered care. The authors reported that, despite nurses' intention to meet patients' needs, care delivery was predominantly centered on routine nursing activities and physical aspects of care (Höglander et al. 2023). The literature suggests that communication practices, including the balance between task‐oriented questioning and patient‐centered approaches such as open‐ended questioning, active listening, and the teach‐back method, may influence patients' engagement in assessment and medication management discussions (Street Jr et al. 2009).

### 4.2 Opportunity

The opportunity to conduct a structured screening and assessment of medication literacy is linked to social influence. Medication management in home care settings is a complex task requiring interprofessional collaboration. The home care setting in Switzerland is fragmented: physicians, pharmacists, and RNs are not usually employed by the same organizations. In their systematic review, Larsson et al. (2022) confirmed that the home care environment was complex, requiring familiarity and cooperation between all the healthcare professionals involved to strengthen interprofessional collaboration (Larsson et al. 2022). The literature showed RNs to be in a central position within interprofessional teams and key to coordinating patient care (Larsson et al. 2022).

Social influence also plays a role within local contexts: at both our study sites, implementation only truly started after one person decided to take the lead. Indeed, this itself had been induced by a study team member's email follow‐up. Constant supervision subsequently helped the pilot project's implementation efforts.

The literature also clearly shows that leadership behaviours that provide strategic direction, address implementation barriers, model evidence‐based practice, and reinforce expected standards are distinct from educational interventions alone (Sebire et al. 2025).

### 4.3 Motivation

As described above, RNs play key roles in coordinating care and in interprofessional collaboration. To fulfil these tasks, RNs must be aware of their social and professional roles and identity. We observed that not all nurses seemed to think of themselves as members of a profession that regularly conducted structured assessments using evidence‐based instruments. They saw this as an activity inherent to the roles of physicians or psychologists rather than those of nurses. This seemed to influence RNs' motivation to conduct structured assessments. However, the literature generally presents medication management as a nursing activity not only involving monitoring, education, identification of medication‐related problems, and communication with prescribers (Mardani et al. 2020), but also assessment (De Baetselier et al. 2020). Philippa et al. (2021) conducted a mixed‐methods research study that concluded in general that nurses must be patient‐focused in order to enhance their professional identity. This might then also have a positive effect on nurses' perceptions of their professional role: Philippa et al. stated that nurses' confidence increased and their professional identity expanded when they applied their nursing knowledge and added skills to their scope of practice (Philippa et al. 2021).

Motivation was also linked to beliefs about consequences. We identified that it was important for RNs to be made aware that using structured assessments benefited patients. If RNs believed that their interventions had positive effects on patients, this could facilitate their willingness to use structured assessment instruments. This was also in line with Jun et al. (2016), who conducted an integrative review and identified internal factors, such as nurses' perceptions and attitudes, as potential barriers to nurses using clinical practice guidelines.

This might be linked to RNs' intentions. Besides RNs' beliefs about consequences, we identified the MELIA instrument's usability as a barrier to RNs' intentions to use it, especially the user manual. The manual's text was perceived as too long to be read on workdays and thus represented a barrier to RNs' intention to use the MELIA. Brooks et al. (2019) found similar results when investigating healthcare professionals' views on using health literacy screening tools. They reported that some RNs perceived using screening tools as burdensome and time‐consuming. Digital solutions could be applied to counter this, as proposed by Leder et al. (2022), whose review of the literature described digital solutions for providing instructions in industrial manufacturing contexts. We suggest that this knowledge could be applied to home care settings, too. Digital solutions enable the use of different ways of presenting instructions that fit individual users' requirements (Leder et al. 2022), including combinations using text, images, video or animation, and augmented reality (Leder et al. 2022).

### 4.4 Study Strengths and Limitations

One of this study's strengths was its use of a multi‐method design, which enabled us to choose matching data collection methods in each of its phases. Another strength was the consistent reference to theory and frameworks. This provided us with more detailed information about the complex issues of implementing the MELIA in home care settings. These results could improve the quality of care provided by home care organizations and may thus improve the safety of older adult home care patients through better medication safety.

The study had some limitations; nevertheless, including its small sample size, predominantly qualitative design, and the potential for selection bias that leads to context‐bound results. Our findings may thus not be generalizable to other home care organizations in different national or international contexts.

### 4.5 Recommendations for Future Research

Future research should focus on how home care organizations change their structured assessment implementation strategies. The MELIA should be used in additional home care organizations to investigate how to promote the implementation of structured screening and assessment instruments for analysing home care patients' medication literacy. The future transferability and scalability of the study's results should also be investigated.

### 4.6 Implications for Policy and Practice

- Nursing curricula should put a greater focus on nurse–patient communication, on providing contextual information about how home care systems in Switzerland operate, and on clarifying role allocation among healthcare professionals, especially when assessing patients' overall health situations, including their medication literacy.
- Policy makers should focus on promoting home care treatment networks that ensure a common understanding of the roles and responsibilities of every healthcare professional involved in medication management in home care settings and on fostering collaborative communication between them.
- Home care organizations seeking to use the MELIA should provide clinical leadership and supervision for its application. Clear organizational structures should be put in place for acting on the assessment's findings, including support for RNs. Professionals in leadership positions should ensure that every RN knows about the positive impacts on patients that can result from conducting structured clinical assessments.

## 5 Conclusion

The present pilot study enabled us to identify the determinants of successful implementation projects for introducing and using new assessment instruments in home care settings. Barriers to the introduction and use of our structured MEdication LIteracy Assessment (MELIA) involved the theme of *capability*, including nurses' deficient communication skills, their lack of knowledge about how to conduct structured assessments, and a lack of knowledge about the responsibilities inherent in their own roles and those of other healthcare professionals. Themes linked to *opportunity* included the challenges of interprofessional cooperation resulting from time constraints, unclear responsibilities, and problems with the usability of the MELIA itself. Themes linked to *motivation* included registered nurses' beliefs about the inherent usefulness of structured clinical assessments and their willingness to read lengthy instruction manuals and conduct structured assessments using evidence‐based tools. Registered nurses' roles in home care settings must be strengthened and clarified to foster the use of structured assessment instruments that benefit patients and increase medication safety.

## Author Contributions

**Samuel Stenz:** conceptualization, methodology, data curation, investigation, formal analysis, visualization, writing – original draft, writing – review and editing. **Carla Meyer‐Massetti:** conceptualization, methodology, validation, supervision, resources, writing – review and editing. **Franziska Zúñiga:** conceptualization, methodology, validation, supervision, resources, writing – review and editing. **Kornelia Kotkowski:** conceptualization, methodology, writing – review and editing.

## Funding

The authors have nothing to report.

## Ethics Statement

The Ethics Commission of Northwestern and Central Switzerland (EKNZ) verified the research proposal and concluded that the study was a quality improvement project and, therefore, did not require ethics approval as it did not fall within the scope of the Federal Act on Research involving Human Beings (Req‐2023‐00741).

## Conflicts of Interest

The authors declare no conflicts of interest.

## Data Availability

The data that support the findings of this study are available from the corresponding author upon reasonable request.

## Appendix 1 Questionnaire

QUESTIONNAIRE TO BE COMPLETED AFTER USING THE MELIA: the Medication Literacy Assessment instrument.

### Part 1: Demographic Data

Please answer a few questions about yourself.

Age:______

Gender: female, male, other, do not want to answer

How long have you been working for a home care organization? ___________________________

Which home care organization do you currently work for?

Spitex XXX Spitex YYY

How often have you already used the MELIA?

It was the first time I had used the MELIA before

### Part 2: Acceptability of the Melia

The following four questions measure how acceptable you find using the MELIA questionnaire for recording home care clients' medication literacy. The questions are intentionally worded in a very similar manner. This similarity helps to ensure the accuracy of our statistics.

*Acceptability of the Intervention Measure (AIM)* (Weiner et al. 2017; Kien et al. 2021)

**Table:**

	Do not agree at all	Do not agree	Neither agree nor disagree	Agree	Totally agree
1. I approve of using the MELIA	①	②	③	④	⑤
2. Using the MELIA appeals to me	①	②	③	④	⑤
3. I like using the MELIA	①	②	③	④	⑤
4. I welcome using the MELIA	①	②	③	④	⑤

### Part 3: Feasibility of the Intervention

The following four questions measure how feasible you find using the MELIA questionnaire to record home care clients' medication literacy. These questions are also intentionally worded in a very similar manner. This similarity helps to ensure the accuracy of our statistics.

*Feasibility of the Intervention Measure (FIM)* (Weiner et al. 2017; Kien et al. 2021)

**Table:**

	Do not agree at all	Do not agree	Neither, nor	Agree	Totally agree
5. Using the MELIA appears to be feasible	①	②	③	④	⑤
6. Using the MELIA seems to be possible	①	②	③	④	⑤
7. Using the MELIA seems to be feasible	①	②	③	④	⑤
8. The MELIA instrument appears to be user‐friendly	①	②	③	④	⑤

### Part 4: Opportunity to Communicate

Please use this space as an opportunity to share your thoughts with us. These can be suggestions for improvement and/or other thoughts on using the MELIA to record home care clients' medication literacy.

## Appendix 2 List of Barriers and Facilitators

BARRIERS TO AND FACILITATORS OF BEHAVIOUR CHANGE IN RNs AND THEIR USE OF THE MELIA (the Medication Literacy Assessment) FOR SCREENING AND ASSESSING MEDICATION LITERACY.

**Table:**

COM‐B	Code (TDF)	Themes (conceptualized as domain summaries)
CAPABILITY	Knowledge	KNOWLEDGE MANAGEMENT: Knowledge management helps to facilitate behaviour change towards the use of the MELIA for screening and assessing patients' medication literacy. RNs' knowledge of medication literacy and how to use the MELIA should be monitored regularly. This helps to test the effectiveness of training courses and identify misconceptions. It also helps to contain misinformation One way to achieve this could be a communication channel or platform that enables RNs to share their knowledge in a timely manner, including the chance to share their experiences of using the MELIA. This channel could help RNs to solve their problems using the MELIA among themselves or enable their colleagues in leadership positions to address problems and provide their support Knowledge management could also help RNs to keep up to date with the latest knowledge. This should also be facilitated by disseminating the present study's results as soon as possible
	Skills	THE BEHAVIOUR (and abilities) OF INDIVIDUAL RNs: The behaviour and abilities of individual RNs can be barriers to the successful use of the MELIA, especially if they perceive the instrument to be a collection of inflexible rules with boxes that need to be ticked instead of using it as a guideline for screening and assessing patients' medication literacy. RNs' ability to empathize with patients can counteract this and be a facilitator of behaviour change. This could also be influenced by individuals' endorsements of the MELIA
		TRAINING and GUIDANCE: Training could be a facilitator of behaviour change towards the regular use of the MELIA for screening and assessing patients' medication literacy. Training should focus on the MELIA's correct usage as well as RNs' ability to explain why using the MELIA is important. This should take place before the MELIA is used for the first time. During training sessions, RNs should be given the opportunity to discuss and learn how to communicate with patients about medication literacy and how to ask questions. Another topic should address the meaning and purpose behind using the MELIA. The effectiveness of training courses should be monitored regularly through a review of whether the MELIA is being used correctly
	Behavioural regulation	Institutional guidance (from people in leadership roles and through communication and reminders) The desired behaviour change among RNs could be positively influenced if using the MELIA were mandatory and supervised by managers. Managers and RNs alike should be reminded to use the MELIA, and this could be supported electronically
	Memory, attention and decision processes	Reminders (guiding processes) and awareness of one's job description RNs may be more likely to use the MELIA if they are made aware that it is an integral part of their duties. This would be facilitated if home care organizations made this clear in advance, including which professionals were tasked with using the MELIA in which situations. This could be facilitated further if RNs were reminded to use the MELIA when it is applicable
		Behaviour can be regulated when it becomes routine Regular use of the MELIA helps to build a routine, and this could facilitate the desired behaviour change
		Individual or personal resentment towards new things or change Change and new things are often rejected. If individual RNs are resistant to innovation, this can be a barrier to the group's behaviour change, especially if those individuals have a strong influence on the opinions and attitudes of other team members
OPPORTUNITY	Environmental context and resources	Internal and external processes and interprofessional collaboration Unclear processes could have been barriers to the desired behaviour change. This included external and internal processes. To counter this, it should be clear who does what and when This is especially important to ensure well‐functioning interprofessional collaboration. This barrier could be overcome if RNs knew when to inform other healthcare professionals about the results of a MELIA and what kind of language to use in their communication. This could be facilitated by having a dedicated RN or manager who can be asked for help if anything is unclear
		Usability (supported by resources and technology) should be as easy as possible The MELIA should be as easy to use as possible. Usability thus becomes a facilitator of the desired behaviour change. Usability includes the ease of use of the instrument itself and organizational factors, such as its availability at the point of care (complicated in paper format) ‐ and its integration into daily practice Integrating the MELIA into the suite of electronic tools and software used by RNs could facilitate its use by making it immediately available to them in their everyday practice. Software could also provide prompts so that RNs use the MELIA, e.g., with reminders linked to previously used assessment instruments, such as Switzerland's interRAI home care assessment system Time Time pressures could be a barrier to using the MELIA. It is therefore important that RNs are allotted enough time to perform the intervention and that they assign the time used to an appropriate budget line or cost centre
	Social influence	Internal social influence Role models could facilitate the desired behaviour change through their influence on team culture. This could be facilitated further if training sessions for using the MELIA were provided for teams rather than for individuals. The opportunity to share experiences can strengthen team culture even further and therefore facilitate the use of the MELIA
		External social influence External social influences, such as other healthcare professionals or patients, could be a barrier to the desired behaviour change, e.g., if other healthcare professionals, like GPs or pharmacists, react negatively to RNs' recommendations resulting from the MELIA. Another barrier would be if patients refused to undergo assessments conducted by RNs. Both cases could be influenced by RNs' professional behaviour and communication
MOTIVATION	Reinforcement	Reinforcing the use of the MELIA through accompaniment and monitoring by a dedicated contact person A dedicated contact person in a leadership position, such as an advanced practice nurse or a team leader, can facilitate the desired behaviour change by monitoring and perhaps even enforcing the MELIA's regular use. This person can influence RNs' motivation.
		Positive reinforcement by praising the correct use of the MELIA Positive reinforcement by appreciation for using the MELIA correctly could facilitate the desired behaviour change
	Goals	Pursuing unified goals helps to motivate teams Having unified goals could facilitate the desired behaviour change by motivating RNs. For that to work, it is important that the goals set are communicated to every team member
	Intentions	The (unconscious) rejection of change New methods leading to organizational change are often rejected. This often unconscious resistance could have a negative impact on RNs' motivation to use the MELIA
	Beliefs about consequences	If RNs believe that using the MELIA leads to benefits for patients, then its regular use might be facilitated RNs could become demotivated if they must spend energy and time using an assessment tool without seeing any obvious return. The potential benefits of using the MELIA should therefore be communicated clearly to RNs. It also helps if users reflect on the instrument's use and the impact it has had.
	Beliefs about capabilities	Empowering leadership culture could help to tackle RNs' fears or worries Fears or worries could be barriers to the desired behaviour change. Staff in leadership positions can influence these by encouraging an environment that allows RNs to express their fears or concerns and provides them with support to cope. This could lead to RNs with greater feelings of empowerment and a belief in their ability to use the MELIA correctly. Empowerment can further be facilitated if RNs receive good basic training on using the MELIA
	Emotion	Individuals' emotions could hinder behaviour change Individual RNs' emotions could act as barriers to the desired behaviour change. This could include the rejection of change and new interventions or fears and worries informed by past negative experiences
	Social or Professional Role and Identity	A clear understanding of one's responsibilities and role identity could facilitate behaviour change A clear understanding of one's own responsibilities and a clear allocation of tasks among the home care team members could facilitate the desired behaviour change. RNs who are not responsible for case management could be involved in this process by assigning them the task of using and promoting the MELIA. This could also lead to the role's increasing attractiveness
